# Accurate Prediction of Severe Allergic Reactions by a Small Set of Environmental Parameters (NDVI, Temperature)

**DOI:** 10.1371/journal.pone.0121475

**Published:** 2015-03-20

**Authors:** George Notas, Michail Bariotakis, Vaios Kalogrias, Maria Andrianaki, Kalliopi Azariadis, Errika Kampouri, Katerina Theodoropoulou, Katerina Lavrentaki, Stelios Kastrinakis, Marilena Kampa, Panagiotis Agouridakis, Stergios Pirintsos, Elias Castanas

**Affiliations:** 1 Department of Experimental Endocrinology, University of Crete, School of Medicine, Heraklion, Greece; 2 Department of Emergency Medicine, University of Crete, School of Medicine, Heraklion, Greece; 3 Department of Biology, University of Crete, School of Sciences and Technology, Heraklion, Greece; 4 Health Center of Gerani, Chania General Hospital, Mournies Chania, Greece; 5 Botanical Garden, University of Crete, Gallos Campus, Rethymnon, Greece; Cincinnati Children's Hospital Medical Center, University of Cincinnati College of Medicine, UNITED STATES

## Abstract

Severe allergic reactions of unknown etiology,necessitating a hospital visit, have an important impact in the life of affected individuals and impose a major economic burden to societies. The prediction of clinically severe allergic reactions would be of great importance, but current attempts have been limited by the lack of a well-founded applicable methodology and the wide spatiotemporal distribution of allergic reactions. The valid prediction of severe allergies (and especially those needing hospital treatment) in a region, could alert health authorities and implicated individuals to take appropriate preemptive measures. In the present report we have collecterd visits for serious allergic reactions of unknown etiology from two major hospitals in the island of Crete, for two distinct time periods (validation and test sets). We have used the Normalized Difference Vegetation Index (NDVI), a satellite-based, freely available measurement, which is an indicator of live green vegetation at a given geographic area, and a set of meteorological data to develop a model capable of describing and predicting severe allergic reaction frequency. Our analysis has retained NDVI and temperature as accurate identifiers and predictors of increased hospital severe allergic reactions visits. Our approach may contribute towards the development of satellite-based modules, for the prediction of severe allergic reactions in specific, well-defined geographical areas. It could also probably be used for the prediction of other environment related diseases and conditions.

## Introduction

Acute type I hypersensitivity (allergic) reactions affect 20% of the world population and 0.1% of cases are severe or even fatal [1,2]. Allergic reactions impose a major (direct and indirect) economic burden to society. For example, in the UK, treatment for asthma and other allergic disorders account for 10% of primary care-prescribing costs [3]. However, in spite of their significant impact on human health, there exists no widely accepted accurate model for their prediction. This might be due to the high diversity of allergens and their disperse spatio-temporal distribution. Allergic reactions have been related to a number of economical (Gross National Product, GNP), biochemical and genetic parameters (lipids, lipoproteins), as well as climatic (altitude, temperature, humidity) and environmental (air pollution) regional conditions [4–10]. However, no single parameter or combination of parameters has been successfully advanced as a predictor of allergic burden, probably due to non-dichotomous (i.e. non-binary) distribution of these variables,. In addition, some of these parameters are difficult to estimate in a day-to-day practice, challenging the establishment of a robust, convenient and efficient model of allergy prediction. Added to this difficulty is the fact that prevalence of allergies does not follow a unique pattern world-wide, with up to 20-fold variations among countries [11].

Meteorological variables (temperature, humidity, rainfall, wind speed and direction), vegetation characteristics, altitude and air pollution conditions have been implicated in the short- and long-term loco-regional distribution of allergic reactions [4–9]. However, their spatiotemporal variability and the different study strategies used for their assessment and validation have led to inconclusive data regarding their cumulative impact in allergy incidence and prevalence. Such studies have helped in our better understanding of the pathophysiology of hypersensitivity reactions [5,7,9] and to the identification of factors related to climaxes of allergic reaction incidence. Nevertheless, the establishment of major global causal effects of these variables in the pathophysiology and frequency of allergic diseases could not be established, especially in view of the impact of genetic and environmental extreme variations in allergy prevalence at broad geographic areas [11]. Therefore, a model describing and/or predicting severe allergic reactions still remains a challenge.

**Fig 1:**
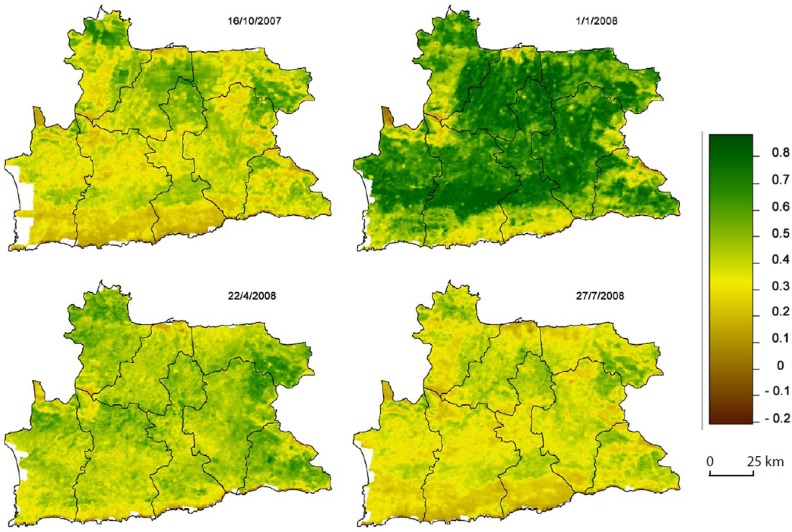
Representative seasonal variation of NDVI measurements for 2007–2008 in the Heraklion-Prefecture. NDVI values follow a 15 or 16 days temporal resolution. Figure was generated using ArcGIS v.8.0 and NDVI values are freely available from the MODIS web (http://modis.gsfc.nasa.gov/).

Recently, through an intense trans- or international effort, the launch of a number of satellites provided the international community with a freely available set of physical and meteorological measurements, which could potentially be used for the description of environmental-related diseases. Such parameters include, among others, temperature measurements and vegetation estimates. The latter has been available through Normalized Difference Vegetation Index (NDVI), a numerical parameter derived from the normalized difference of near infra-red and red reflection measurements (a representative seasonal variation of NDVI measurements for 2007–2008 in the Heraklion-Prefecture is presented in Fig. 1, drawn with the ArcGIS v.8.0 program and freely availlable NDVI data from the MODIS web of NASA (http://modis.gsfc.nasa.gov/)). Therefore, NDVI provides a global measurement of live green vegetation, as chlorophyll absorbs visible radiation and reflects near infra-red [12].

As measurement and estimation of global live vegetation relates to plant development and integrates a number of parameters including rainfall, wind, humidity and wind parameters, we questioned whether such a parameter, combined with meteorological data could be sufficient to describe and predict the incidence of allergic reactions severe enough to necessitate a hospital visit, in well-defined geographic areas. Our data suggest that, at a loco-regional basis, such an approach is valid, providing a tool for the development of satellite-based prediction modules for severe allergic reactions and probably for other environmental-linked diseases and conditions.

## Materials and Methods

### Ethics statement

This study was approved from the Research and Ethics Committees of the University Hospital of Heraklion and the General Hospital of Chania. Since the collected data were obtained through institutional statistics and no personal patient data were recorded, the Research and Ethics committees of both hospitals did not request formal patient consent for the proposed study.

### Study Area

Crete is the largest island of Greece and the fifth largest in the Mediterranean Basin, with a typical Mediterranean climate characterized by hot and dry summers and mild, cool and wet winters. The island climate is representative of the dry Mediterranean environments of Southern Europe and the Mediterranean Basin. In this study, we focused in the two most populated regions of the island, which are also the main tourist destinations, namely Heraklion and Chania. Heraklion Regional Unit is located at the center of the island, divided in 8 municipalities. The landscape is characterized by a lowland and semi-mountainous terrain in the center, with altitudes lower than 1000m and lowlands in the North and South parts. The climate is typically Mediterranean, with warm, dry summers in the lowlands, and cooler, rainier conditions in the mountains. The metropolitan area of Heraklion, located on the northern coast of the island, reaches 173450 inhabitants. Chania Regional Unity is located at the western part of the island, divided in 7 municipalities. The landscape of the area is characterized by a main mountain in the southeastern part (∼2500 m), with semi-mountainous terrain on the southwest and lowlands in the north. The climate of this study area is similar to Heraklion, but with higher annual precipitation rates. The metropolitan area of Chania, equally located on the northern coast, has 55838 inhabitants.

### Patients’ data

Two separate sets of data were collected for the Heraklion area: (1) Two cohorts of Emergency Department visits for allergic reactions at the University Hospital of Heraklion for the periods September 2007 to August 2008 and September 2009 to August 2010. All cases of severe allergic reactions (hives, angioedema and asthma exacerbations) were retrospectively identified from the Emergency Department records. To be included in the study, each case had to be signed by a physician. Only cases of unknown etiology (i.e. cases where an allergy exacerbating factor could not be identified during the patient’s visit to the Emergency Department) were included in the study. Allergic reactions to food, animals or medications were not included. Regarding patients with asthma, cases that were clinically attributed to infection were also not included in the study. An allergic reaction was considered severe if any of the following occurred: (i) decision to treat the patient with adrenalin or intravenous antihistamines/corticosteroids or inhaled bronchodilators/corticosteroids, (ii) decision to observe the patient for at least 3 hours or (iii) decision to admit the patient for further treatment/observation. (2) Results of referred patients for specific IgE measurement (measured on a Siemens Immulite 2000 apparatus, Siemens Hellas Diagnostics, Athens, Greece) for the same time periods. Only the subset of patients testing positive for animal, plant, fungus and dust-related allergies was used in the study. Data were exported anonymously from the LIS of the University Hospital database.

For the Chania area, emergency department visits for allergic reactions during September 2007 to August 2008 were collected, based on the same principles described above.

### Environmental parameters

The dataset of environmental parameters included nine variables: NDVI, temperature (mean, maximum and minimum), rainfall and wind (speed, direction, deviation from northness and eastness). Altitudinal and topography data were obtained from the Digital Elevation Model of the study area (http://srtm.csi.cgiar.org), municipality borders were accessed from the Information Communication Technology and Documentation Center of the Region of Crete, while population data for each municipality and municipal district were provided by the Hellenic Ministry of Interior, based on the 2011 population census. Meteorological data were retrieved from the Hellenic National Meteorological Service (HNMS, Heraklion: Long. 25°10&rsquo;0"/Lat. 35°19&rsquo;0"/Alt 39m, Chania: Long. 24°7&rsquo;0"/Lat. 35°29&rsquo;0"/Alt 150m). Regarding rainfall, wind speed, temperature mean value, temperature maximum and minimum values, the 15 daily measurements, collected from local HNMS stations for each period applied in the study were used to calculate a mean value (14 daily measurements were used for February and 16 day measurements for months that have 31 days). Taking into consideration the cyclic character of the wind direction that is counted in degrees, statistical artifacts could appear (eg 1° and 359° have an actual 2° difference but the calculated difference is 358°). We therefore followed the common practice to incorporate it as eastness and northness using the sin and cos of the angle respectively.Normalized Difference Vegetation Index data, which describe the normalized difference of near infra-red and red reflection measurements and provides a global measure of live vegetation, as chlorophyll absorbs visible radiation and reflects near infra-red [12], were retrieved from TERRA (EOS AM) satellite (http://modis.gsfc.nasa.gov), with the following characteristics: sensor MODIS TERRA, temporal resolution 15 days (14 daily measurements were used for February and 16 day measurements for months that have 31 days), range 250 m, missing data none. For the spatial pattern of NDVI in the Heraklion-Prefecture, representative pictures of seasonal variation for selected dates within the period 2007–2008 are provided in Fig. 1. The satellite-based Land Surface Temperature (LST) measurements were also obtained from http://modis.gsfc.nasa.gov, with the following characteristics: sensor MODIS TERRA, temporal resolution 1 day, range 1000 m. MOD11A1 applies the split-window LST algorithm, optimal for separating ranges of atmospheric column water vapor and lower boundary air surface temperatures into tractable sub-ranges. Per-pixel temperature values obtained for the city of Heraklion were then transformed into the proper day-interval mean and maximum LST, in accordance to HNMS data. Therefore, the mean values of each environmental factor (defined based on the municipality borders) per half-month were used as predictors in the model development process. The geographical presentation of our data has been compiled using ArcGIS v.8.0 program (ESRI, Redlands, CA).

### Data analysis

Emergency department visits data were analyzed on a half month interval basis and statistical modeling was conducted with the R statistical software [13], based on Generalized Linear Modeling, with binomial error distribution [14]. Specifically, if Yi is the count of emergency department visits for half-month interval i, we assume that *Υ_i_* ∼ binomial(*N*, *p_i_*), where N is the total population of the area (the city of Heraklion for the Heraklion data set and the city of Chania for Chania data set) and pi is the proportion of that population that visits the emergency department on half-month interval i. In other words, the number of "successes" for the binomial distribution is equal to the recorded emergency department visits, while the number of "trials" equals the total population. The unknown probability pi was then estimated by the model using a logit link function: $\text{log}\;\left(\frac{p_{i}}{1-pi}\right)=X\beta$, where X is the matrix of environmental variables and β is the vector of the corresponding covariates. Squares and all pairwise variable interactions were also included, to allow for non-linear relationships. The model was developed through a bidirectional stepwise procedure, starting from only the intercept, based on optimization of Akaike Information Criterion (AIC) [15], considered as one of the most appropriate model selection criteria [16]. In addition, as the stepwise algorithm may ignore some models that can reach an increased prediction level, eight “model sets” of a predefined number of variables, including base variables (1 to 8), their squares and all possible combinations were constructed (i.e. model 1 included only one base variable and its square, model 4 included 4 base variables, their squares and all possible interactions, model 8 included all base variables, their squares and all possible interactions). These models underwent a backwards elimination procedure (for models with less than four variables) and a forward selection for models with four variables or more, based on AIC minimization. Finally, we compared our models to a null model, incorporating the seasonality of severe allergic reactions according to the emergency department collected data. The whole procedure was rerun individually for each dataset.

The predictive accuracy of all selected models was assessed via a leave-one-out cross validation procedure, where each one of the 24 half month intervals was left out and subsequently estimated by the model trained using the other 23 intervals. The metric used for the assessment was Mean Absolute Error, defined by the equation: MAE = 1/n ∑_i (|f_i-y_i)/y_i), where n is the number of samples (i.e. the 24 half month intervals in our case), *y*_i_ the observed count in sample *i* and *f*_i_ is the predicted count for the same sample. In all cases of within-sample measures of performance, Spearman’s rho was selected instead of Pearson’s r, because we were interested in monotonic relationships rather than strictly linear ones.

## Results

### NDVI and temperature are sufficient to construct a prediction model for severe allergic cases at a local scale

**Fig 2:**
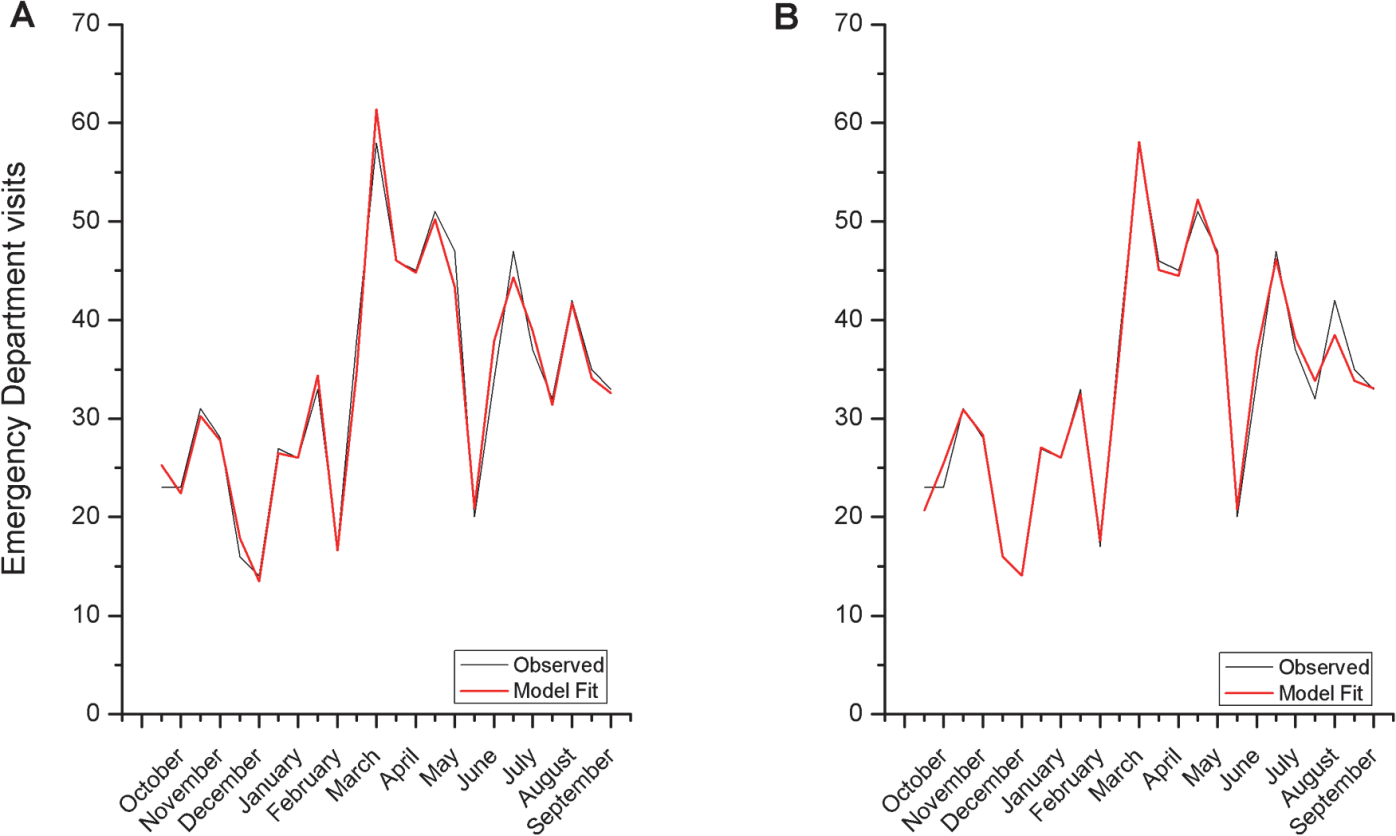
Graphical presentation of the frequency of emergency department (ED) visits at the Heraklion metropolitan area (solid line) and model fit of the NT Model, that is derived from NDVI and temperature (dotted line) (A) and the SAT Model Assemblage, from NDVI and satellite collected Land Surface Temperature (B). See Material and Methods for the development of the models. It should be noted that while the raw output of the models is pi, i.e. the proportion of the total population that performed ED visits, results are presented as counts (pi × N), for ease of interpretation in the Fig. 2 and subsequent Figs. 3–4.

Emergency department visits in 2007–8 (803 patients, Fig. 2A) shows that the major allergic burden occurs during spring, with lower incidence during the winter and summer time-periods. A generalized linear fit (Table 1) of the nine environmental parameters (NDVI, temperature (mean, maximum and minimum), rainfall, mean and maximum wind speed, and wind direction, measured as deviation from eastness and northness (see Material and Methods)) revealed that a model incorporating only NDVI and temperature data (mean and maximal temperature for the reporting period, NT model in Table 1) minimizes the Akaike Information Criterion (156.2), which is not further significantly improved by the addition of other variables and provides the smallest mean absolute error (0.057). Interestingly, in this model, a power term (ndvi^2^) is also significant, as are the temperature terms (mean and maximal temperature interactions), which is suggestive of a non-linear relationship of the variables with allergic reactions burden. A graphical representation of the observed and predicted data is shown in Fig. 2A (Spearman’s rho = 0.9952147, p<0.0001). It should be noted that while the raw output of the models is pi, i.e. the proportion of the total population that performed ED visits, results are presented as counts (pi × N), for ease of interpretation, in Fig. 2A and subsequent Figures. Interestingly, replacement of ground- with satellite-temperature measurement also provides a significant model fit (Fig. 2B, Spearman's rho = 0.9902112 p<0.0001), slightly inferior to ground temperature measurements. In order to further validate our model, we performed an exhaustive screening of all possible terms related to the base variables of NT with the R package glmulti [17]. All models that gave an AIC within 2 units of the best AIC were kept and leave-one-out cross-validation was performed. Analysis revealed that NT model represents the best estimate of our data, suggesting that NDVI and temperature are the keystone environmental parameters for allergy burden estimate at discrete geographical areas.

**Table 1:** Description of models used for allergy description.

Number of base variables in model	Null Model	1	2	3 (NT)	4 to 8	9
AIC	170.0261	191.6104	169.2	**156.2062**	154.7354	156
MAE	0.2398636	0.2693172	0.221505	**0.05704021**	0.09861414	0.1198496
(Intercept)		−9.245228	−10.820534	3.32×10^2^	−9.8488923	−7.772586
Ndvi				−**1.08×10** ^**3**^		−3.527386
Tmean			−0.631477	−5.11	−0.7819236	−0.815993
Tmax		0.034927	0.564828	−25.5	0.6417735	0.598364
Rain					−0.0067363	−0.008827
We					1.6234941	
ndvi^2^				**1.03×10** ^**3**^		
tmean^2^				−1.62×10^-1^		
tmax^2^			−0.013613	4.73×10^-1^	−0.016637	−0.015449
tmax× ndvi				5.91×10^-1^		
tmean× tmax			0.017759	7.60×10^-1^	0.0220229	0.021767
tmean× tmax^2^				−**2.04×10** ^**–2**^		
tmean^2^× tmax^2^				**1.90×10** ^**–4**^		
ndvi× tmax^2^				−6.79×10^-1^		
tmax× ndvi^2^				−39.3		
tmax^2^× we					−0.0016847	

In the upper part of the Table the Akaike Information Criterion (AIC) and the Mean Absolute Error (MAE) are presented. In the lower part, the corresponding coefficients for the different retained variables for each model are shown. Significant values for the retained model are shown in bold. Abbreviations: ndvi, Normalized Difference Vegetation Index; tmean, mean daily temperature; tmax, maximum daily temperature; rain, daily rainfall; we, wind eastness. Squares of each factor are represented with the ^2^ symbol, interactions are represented with the × symbol, in column 1. For the retained NT model (in bold) the most significant retained parameters are shown in bold characters.

### Predictive ability of the NT model

**Fig 3:**
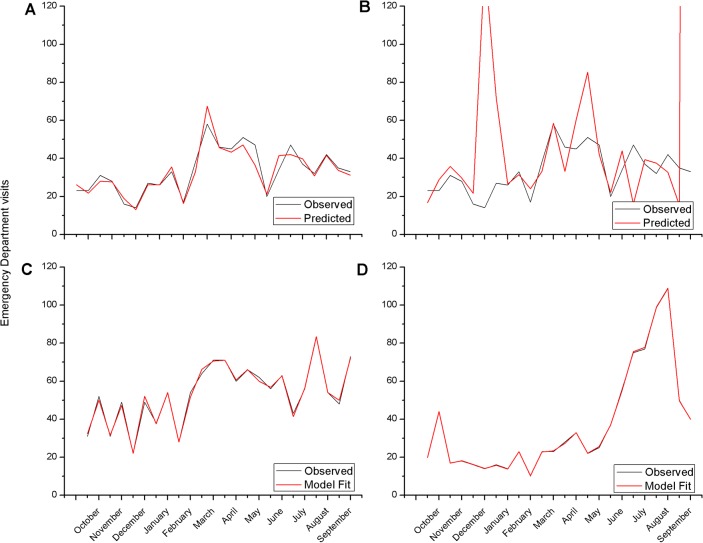
Upper Panels. Predictive value of the retained NT model. Employing a leave-one-out procedure, we attempted to identify whether our models could predict the frequency of emergency department (ED) visits in the metropolitan area of Heraklion. The predictive ability of the NT model, taking into account the ground temperatures (A) was very good, while the SAT model (B), integrating satellite temperature measurements was less performant. Periods refere to half month time-intervals, with the first referring to September 1–15 2007. Lower Panels. Validation of the NT model for severe allergy prediction. In order to validate our NT Model we analyzed a second data-set for the Heraklion metropolitan area (2009–2010) using the same model averaging technique and using the same environmental/meteorological parameters (NDVI, temperature; NT model). Development of the new NT model, based on the same base variables presented a very good model fit both at the Heraklion (C) and at the Chania area (D).

The above data suggest that severe allergic reactions, necessitating a hospital visit, can be accurately described by a small set of environmental parameters (NDVI-temperature), at a local scale. In an attempt to validater and explore the predictive ability of our models, we employed a number of statistical and observational procedures:

1. A leave-one-out procedure was applied, aiming in estimating expected allergic burden half month earlier (Fig. 3A). This half month model resulted in an excellent extrapolation of allergic burden (Spearman's rho = 0.9619319 p<0.0001), comparable to the initial model (compare Figs. 2A and 3A). However, in this procedure, satellite temperature data could not accurately predict allergic reactions distribution (Fig. 3B). This could be due to methodological problems, such as the transformation of IR-radiation measurements to temperature values, because the geostationary satellite is located at a 0° latitude and a major distortion for the island of Crete occurs leading to acquisition of less accurate values.
2. The robustness of the NT model was further validated by the use of the same procedure to analyze a second data-set of allergies for the Heraklion metropolitan area, two years apart of the initial dataset (i.e. 2009–2010 data), in order to verify its long-term temporal validity. Analysis of this second period also provided a very good fit, based on the same parameters (Fig. 3C, Spearman's rho = 0.9908552 p<0.0001).
3. The spatial validity of the model was tested by analyzing a 2007–8 dataset from the Chania area. The prevalence of serious allergy cases presents a completely different pattern in Chania, with a very high incidence of cases in summer months. However, the NT model, adjusted for the Chania area, accurately described the data also (Fig. 3D, Spearman's rho = 0.9986948 p<0.0001, S4 Table).

These results suggest that, at a local scale, NDVI and ground temperature can accurately describe severe allergic reactions, validating our initial hypothesis.

### NT model can accurately describe allergic cases based on RAST measurements

The two hospitals included in the present study are reference centers for more extensive geographical areas, beyond Heraklion and Chania metropolitan locations. At the University Hospital of Heraklion, an Allergy Department exists, which, together with the ORL and Pediatrics Departments, is the reference center for allergies for the whole Region. Therefore, the Immunology-Endocrinology laboratory of this hospital is the reference center for immunoradiometric specific IgE measurements of allergens in the sera of refered patients (RAST tests). In an attempt to explore the validity of the retained NT model to less severe allergic cases (those with positive RAST tests) we have retrieved from the LIS of the hospital all cases of allergies for the reported period (August 2007-September 2008). Only the subset of patients with positive animal, plant, fungus and dust-related allergies was retained for analysis. The resulted dataset was analyzed with the same generalized linear model as above, independently from the analysis of emergency department data. Here, a spatial parameter at the municipality-level (8 municipalities of the Heraklion area) was also introduced as a confounding variable, together with the temporal positive cases at a month-level. This fractionation of data was chosen since based on our experience a patient who had an serious allergic reaction and is expected to have a positive IgE test, is usually referred for such studies within 10 to 15-days after the serious allergic event. The freely available software WinBUGS [18] was used for this analysis.

**Fig 4:**
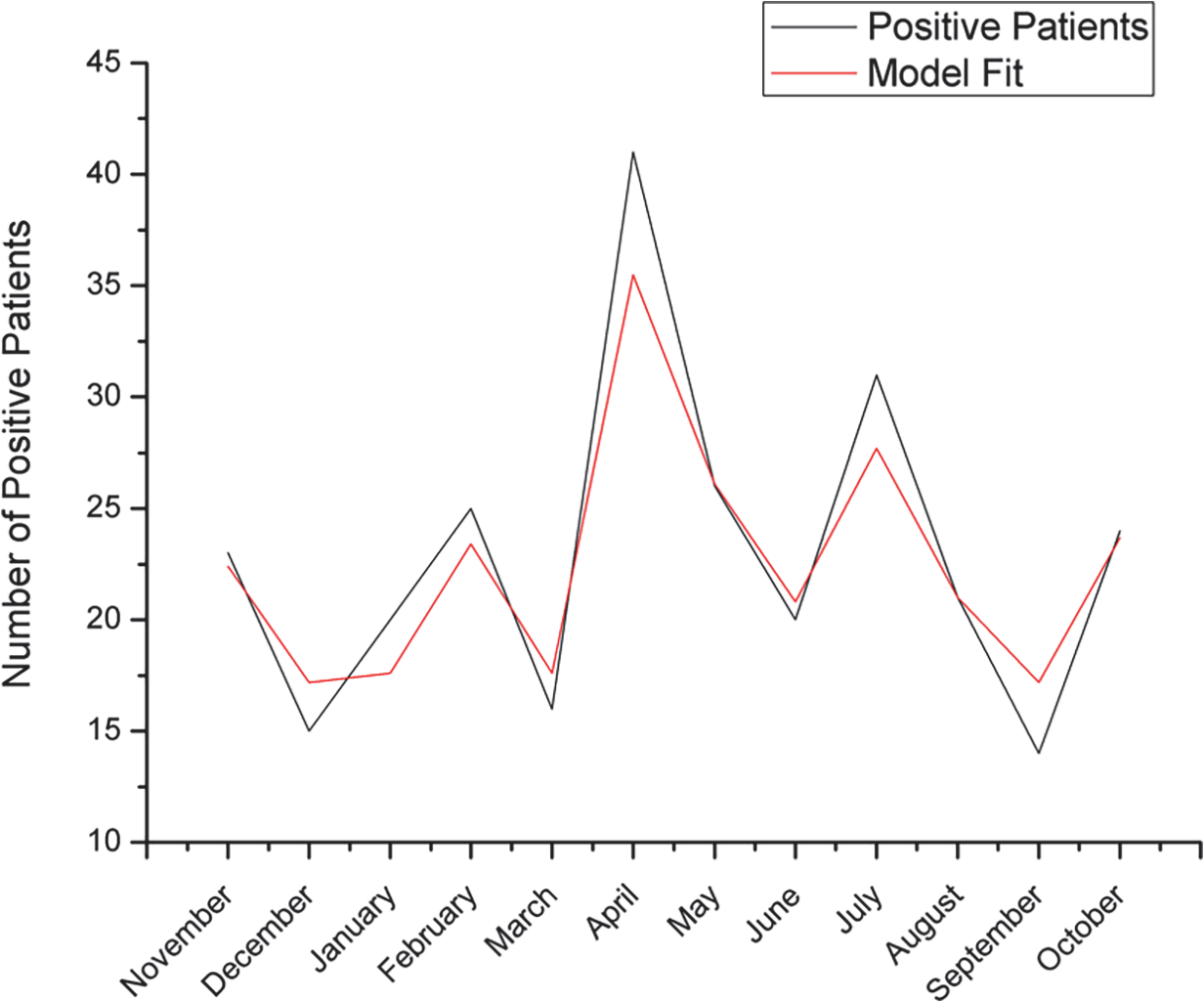
Graphical presentation of the frequency of patients from the regional area of Heraklion, positive for specific IgE, refered to the University Hospital of Heraklion (solid line) and model fit of the NT Model (dotted line).

Data analysis revealed that the same model (NDVI, temperature) accurately describes the spatio-temporal distribution of specific IgE measurements, in the whole geographic area of Heraklion district (Spearman’s rho = 0.9685547, p<0.0001, Fig. 4), suggesting that the NT model is a good predictor of allergies at a local scale.

## Discussion

Allergic reactions may evolve from simple irritating symptoms to life threatening cases [1,2]. However, according to the best of our knowledge, in spite of an intense international effort, a simple accurate model of severe allergic reaction description and prediction, incorporating environmental parameters, has not been reported until now. The lack of such a model might be due to a number of elements inherent to allergic reactions: (i) problems of scale (from local to global) [11], with some variables not fitting proposed models outside the barriers of local scale. Indeed, the majority of models, until now, try to integrate global variables as predictors and/or initiative causes of allergies; (ii) methodological problems, as studies follow a divisive scheme, centering on specific variables, leaving behind the cumulative effect of non studied variables. In addition, the majority of studied variables represent a continuum and discrete binary states are not always easy to settle; and (iii) compatibility problems, as huge effort is needed for the establishment of exploitable data sets for the majority of the variables.

Here, in contrast to previous strudies, we have focused on the description and the prediction of severe allergic reactions at a local scale, in two metropolitan areas of a typical Mediterranean environment. Our goal was to provide a simple model, based on freely available data, in order to describe and predict (within a half month window-frame) a possible severe burden of allergic reactions. Such a model could be of value for health administrators, health authorities and patients and could lead to a severe decrease of allergy-related health cost, in addition to an improved health status of involved individuals. We have focused therefore to available meteorological and vegetation data, obtained from civil satelites. Indeed, in recent years, free satellite data have been made available, through combined multinational or international programs. Among these, temperature measurements are provided, although the geographical coverage is far from being adequate, as was also found in this study. In addition, NDVI satellite data provide integrative global vegetation estimates, with an adequate geographical coverage.

Allergies have been reported to relay heavily on a number of climatic (altitude, temperature, humidity) and environmental (air pollution) regional conditions [4–9]. Here, our idea was to integrate all these masurements through the analysis of the Normalized Difference Vegetation Index (NDVI). NDVI describes the normalized difference of near infra-red and red reflection measurements, providing a global measurement of live green vegetation, as chlorophyll absorbs visible radiation and reflects near infra-red [12]. Therefore, NDVI integrates different species and pollen spread phenological status and variability of vegetation, the vegetation greening, the land-cover and land-use types and the vegetation changes as a result of interactions between plants and animals. In addition, we have retained temperature measurements, both ground and satellite based in our analysis, together with other meteorological data. Our results show that NDVI and temperature (NT model) are sufficient to accurately describe and predict the frequency of allergic reactions, severe enough to necessitate a hospital visit, at a local scale, even for small time-series of one year. Incorporation of wind, humidity and rainfall data did not increase the accuracy of the model. We have attributed this robustness to the technical nature NDVI, which, as described above, may integrate the information of these additional meteorological parameters, thus downgrading the need for the collection of additional data. It is to note that a similar approach, using stellite data of NDVI values has been previously used to determine the onset of the flowering and pollen season for several plant species [19,20]. Furthermore, in a recent work, Fuertes et.al. reported a significant association between the combination of data from vegetation (with the use of NDVI values) and temperature exposure with intermittent rhinitis symptom in children [21]. They aso reported that this association was stronger when centered data from small countries were used. This supports the validity of our approach, since we have used NDVI values for much smaller regions.

Therefore, with the prospect of using the satellite data available, our approach may also contribute towards the development of satellite-based methods, for the prediction of severe allergic reactions and probably of other environment-related diseases and conditions.

Our model was proven robust, as it describes correctly severe allergic cases in the same region two years later and in another area of the island of Crete, Chania that present with a completely different allergy incidence pattern that could be attributed to the different vegetation, and thus the different allergens present in this part of the island. The same parameters may also describe the spatio-temporal distribution of IgE-positive cases, suggesting a relationship between vegetation, temperature and airborne allergen induced allergic reactions. Interestingly, our model could also accurately predict, at a half month interval, the number of severe allergies, necessitating an emergency hospital visit. Therefore, the validity of the NT model was proven in two areas with a typical Mediterranean climate. Whether the same or another limited sub-set of meteorological data will describe allergic reactions in other areas, with a different climate, remains to be tested.

In spite of its descriptive and predictive ability, our study has a number of limitations: (i) It was performed retrospectively, in two geographic locations of the same region. Future additional prospective studies should be performed in other regions, with different climatic characteristics in order to fully validate our approach. (ii) The predictive accuracy of our study is largely based on within-sample measures of performance (Spearman’s rho). In order to account for a possible bias with the use of AIC, leave-one out cross-validation and application of Mean Absolute Error was also included in the study. (iii) Given the small data sets, overfiting is a limitation for incorporating more variables. (iv) The data used for our study may not provide enough information for the identification of asthma exacerbations that may had been triggered from viral infections. However, asthma cases in this study accounted for less than 10% of cases. (v) The algorithms used for NDVI pixel clustering as well as the physical basis of this important novel measurement may affect its precision to express different vegetation types, differences in background reflectance (particularly important in sparsely vegetated areas), variations in vegetation coverage and climate induced phenological shifts [22]. For example, a specific NDVI value in our study areas may represent a different set of allergy related vegetation factors compared to the same NDVI value in an area with a different climate and a different flora. Further studies are needed in order to understand if such effects may limit the general application of our model. Finally (vi) our study did not aim to confirm that the allergic reactions are to specific environmental allergens since such detailed data were not included in original design of the study.

A number of further studies could improve the effectiveness of this approach with the development of an expert-intelligent system for the prediction of allergy burden based on automatically generated data of: (i) lists of emergency department cases with ICD-10 codes related to serious allergic reactions from hospital electronic records, (ii) meteorological temperature predictions, (iii) NDVI predictions which are possible through mathematical approaches [23,24] (iv) methodological improvements in available satellite meteorological data instead of derived data of *in situ* measurements.

In conclusion, our approach shows that a small set of easily available environmental parameters (NDVI, temperature), could accurately describe and predict the trend of severe allergic reactions incidence. We suggest that following this methodology, each region, especially a region where data of long time-series about allergies are not available, could have its own limited set of parameters that could provide proper spatiotemporal prediction models of serious allergic reactions prevalence. Such an approach could lead to the identification and mapping of homogeneous “allergioregions” based on factors involved in the description and prediction of allergies and can prepare local and regional health systems and affected individuals (e.g. via an online allergy-risk indicator) for major peaks of serious allergic reactions that may lead to emergency department overcrowding.

## Supporting Information

S1 Table Data used for the development of the models in Heraklion metropolitan area for 2007–8 and 2009–10. (XLSX)

S2 Table Data used for the development of the models in Chania metropolitan area (2007–8). (XLSX)

S3 Table Data used for the development of RAST model (2007–8), presented in Fig. 4. Data are presented per municipality of the Heraklion Regional Unit (presented in Fig. 1).
(XLSX)

S4 Table Covariate estimates of the NT model for the three data sets (Heraklion 2008–2009, Chania 2008–2009 and Heraklion 2009–2010). Abbreviations and symbols are the same with Table 1 of the main text.
(DOCX)
