# Correction: Accurate Prediction of Severe Allergic Reactions by a Small Set of Environmental Parameters (NDVI, Temperature)

**DOI:** 10.1371/journal.pone.0127604

**Published:** 2015-04-29

**Authors:** George Notas, Michail Bariotakis, Vaios Kalogrias, Maria Andrianaki, Kalliopi Azariadis, Errika Kampouri, Katerina Theodoropoulou, Katerina Lavrentaki, Stelios Kastrinakis, Marilena Kampa, Panagiotis Agouridakis, Stergios Pirintsos, Elias Castanas

The following information is missing from the Funding section: This work was partially funded by the European Union Programs Regional Potential/Translational Potential Grant 285948 (to GN and EC).
