# Supplementary material for: Accurate Prediction of Severe Allergic Reactions by a Small Set of Environmental Parameters (NDVI, Temperature)
Source: PLoS One. 2015 Mar 20;10(3):e0121475. doi: 10.1371/journal.pone.0121475 (PMC4368791; doi:10.1371/journal.pone.0121475)
Supplement: S4 Table — Abbreviations and symbols are the same with Table 1 of the main text. (DOCX) [file pone.0121475.s004.docx]

**Table S4**. Covariate estimates of the NT model for the three data sets (Heraklion 2008-2009, Chania 2008-2009 and Heraklion 2009-2010). Abbreviations and symbols are the same with Table 1 of the main text.

|  | HC | CC | HC |
| --- | --- | --- | --- |
| (Intercept) | 331.9 | 305.8 | 31.86 |
| tmean | -5.108 | -2.733 | -3.083 |
| tmax | -25.49 | -24.25 | -5.647 |
| ndvi | -1084 | -901.4 | 71.5 |
| tmean^2^ | -0.1616 | -0.07869 | -0.3456 |
| tmax^2^ | 0.4728 | 0.4175 | 0.01228 |
| ndvi^2^ | 1029 | 656.4 | -90.22 |
| tmax× ndvi | 0.7604 | 0.4177 | 0.8913 |
| tmean× tmax^2^ | 59.05 | 58.1 | -2.609 |
| tmean× tmax | -0.02043 | -0.01151 | -0.01539 |
| tmean^2^× tmax^2^ | 0.00019 | 0.000105 | 0.000171 |
| ndvi× tmax^2^ | -0.6793 | -0.6741 | -0.0245 |
| tmax×ndvi^2^ | -39.3 | -32.43 | 3.996 |
